# A Unique Way to Axe the Fax Through Using Business Automation Workflow to Expedite eReferral Adoption, Bridging eReferral, and Fax: Proof-of-Concept Study

**DOI:** 10.2196/62983

**Published:** 2025-06-30

**Authors:** Zhigang Tian, Kayla Wierts, Lirije Hyseni, Beth Gerritsen, Kim Lynch, Russell Buchanan, Mohamed Alarakhia

**Affiliations:** 1 Amplify Care, 235 The Boardwalk, Suite 301, Kitchener, ON, N2N 0B1, Canada, 1 5198850606; 2 Institute of Health Policy, Management and Evaluation, Dalla Lana School of Public Health, University of Toronto, Toronto, ON, Canada; 3 Department of Family Medicine, McMaster University, Hamilton, ON, Canada

**Keywords:** eReferral, business automation workflow, fax, referral, electronic referral, electronic fax, fax failure, interoperability, axe the fax

## Abstract

**Background:**

It is estimated that 88% of Ontario physicians still use fax technology to share patient information. Transitioning to electronic referral (eReferral) has been shown to have numerous benefits, but the major barrier to adoption of eReferral is the need for both sending and receiving clinicians on the same platform to enable information sharing. The traditional onboarding process takes time and effort. An innovative method is required to improve eReferral adoption by bridging the gap between eReferral senders and fax referral receivers.

**Objective:**

This study aimed to explore the technological feasibility of leveraging a business automation workflow (BAW) platform to connect the digital (eReferral) and nondigital referral platform (fax), enabling eReferral senders to send referrals to fax receivers, thereby improving the clinician experience.

**Methods:**

An eReferral via eFax solution was developed and evaluated on the BAW platform to connect the eReferral platform and the clinicians using fax. A selected number of fax receivers were identified and enabled on the eReferral platform as eFax receivers. Sending clinicians initiated eFaxes through the familiar eReferral workflow, with eFaxes transmitted to BAW and delivered to the target receiver via fax. Retry and reminder logic were built to improve the user experience. If the eFax failed after all retries, a message was sent to the sending clinician through the eReferral platform explaining the failure reason. The appointment information was entered into the eReferral platform by the sending clinicians to trigger patient email notifications. Surveys and focused interviews were conducted to collect clinicians’ feedback.

**Results:**

From May 2022 to December 2023, 224 eFax receivers were enabled on the platform, processing 4504 eFaxes for 4132 unique patients and 843 unique senders across the province. Nearly 70% (3137/4504) of patients consented and received email notifications; 19% (875/4504) received appointment details after manual entry in the eReferral platform. On average, eFax referrals contained 5.6 pages, with a minimal 0.7% exceeding 30 pages. Initially, fax service retries were disabled to observe delivery error rates. This resulted in a 37.7% (1023/2712) fax failure. However, after implementing new retry logic in March 2023, the failure rate dropped significantly to 9.9% (304/3082), and 98.7% (2770/2806) of eFaxes were successfully delivered after automatic retries. Clinician feedback revealed a positive impact on sending clinicians’ experience, maintaining their familiar workflow while accommodating fax-reliant receivers who can gradually transition to eReferral at their own pace.

**Conclusions:**

This project demonstrates the potential of the BAW platform to bridge the gap between fax and eReferral systems. It minimizes disruption for sending clinicians while allowing fax receivers to incrementally adopt the new platform. This technology can significantly expedite eReferral adoption by reducing the reliance on receiving clinics to adopt eReferral, ultimately enhancing the experience for both clinicians and patients.

## Introduction

When a medical referral is necessary in the Canadian health care system, the sending clinician selects the most appropriate receiving clinician and forwards the patient’s relevant information through fax or other digital technologies, like an electronic referral (eReferral) system [1]. eReferral simplifies the referral process by enhancing communication between primary care clinicians and specialists or organizations, enabling quick and secure referrals to be sent and received through an electronic platform. The eReferral system has been proven to be more secure, can reduce wait times, improve tracking, and lower costs [2,3]. eReferral has also demonstrated the ability to improve patients’ experience [4]. To relieve physician burnout, the Ontario Medical Association recommended enabling electronic information sharing between clinicians to reduce the administrative burden and free up more time to provide patient care [5].

Surprisingly, fax machines are still widely used in health care globally. Fax technology is the lowest common denominator when it comes to physicians who are not working on the same electronic medical record (EMR) systems to share information. It is estimated that around 75% of medical institutions in the United States still rely on faxing for communication [6]. In the United Kingdom, despite the National Health Service’s (NHS) call in 2018 for a complete phase-out of fax use by April 2020 [7], 1 in 6 NHS trusts was found still using fax machines in 2023 [8,9].

The 2021 National Survey of Canadian Physicians indicated Ontario ranked the first in the percentage of physicians sharing information electronically, with 39.9% of the respondents using eReferral to request or receive care from specialists or other physicians for their patients [10]. Although significant progress has been made with eReferral in Ontario, 88% of doctors still have to use fax technology to share patient information [5]. Doctors who already adopted eReferral technology cannot completely move away from the fax technology and have to maintain duplicate workflows to be able to refer to receivers who have not adopted eReferral yet. This has resulted in a significant admin workload for the sending clinicians. In 2023, the Ontario government announced that fax machines would be phased out with digital communication alternatives at all Ontario health care clinicians within the next 5 years in the document, entitled Your Health- A Plan for Connected and Convenient Care [11].

One of the key barriers to eReferral adoption is peers not using eReferrals, the need for both sending and receiving clinicians to have a linked health information system through a shared EMR or an independent eReferral platform [1,2], sometimes also referred to as a “chicken-and-egg” problem. Onboarding the sending and receiving clinicians to the same platform takes time and requires enormous change management support [12]. In a hybrid environment where some receivers adopted the eReferral platform and others are still using fax machines, sending clinicians have reported experiencing frustration over the management of a duplicate workflow in selecting appropriate technologies to prepare and submit the referrals to support patient care.

A new and innovative solution was designed using the business automation workflow (BAW) platform as middleware to bridge the gap between the eReferral senders and the fax receivers. This helps explore the technological feasibility of leveraging the BAW platform to connect digital and nondigital (fax) referral platforms to improve the current referral experience, especially for the sending clinicians. The Amplify Care team (previously eHealth Centre of Excellence) started a Proof-of-Concept (PoC) project in 2022 called eReferral via eFax to better understand using a BAW platform to support managing fax-based referrals by leveraging the existing digital referral platform, to measure fax failure risks and to improve the short-term fax failures and retries management, and in the long term, to support migrating away from fax-based referrals. Eventually, the solution will help expedite the eReferral adoption.

## Methods

### Overview

The design of the solution tried to minimize the impact on the existing workflow for both referral senders and receiving sites so that senders can manage their referrals on one single referral platform, with no need to switch between eReferral and fax workflow and have a seamless experience. At the same time, the receivers can maintain their current workflow to receive the referral on their fax machines. With the new technology implementation, senders should be able to send referrals to a larger group of receivers including those who are still using fax machines, while the fax receivers will start to be exposed to the eReferral technology and this should accelerate their eReferral adoption.

### Platform

The BAW platform has the key capability to receive and deliver information through standard Application Programming Interfaces and it also supports workflow customization based on clinical needs to help automate the tasks and gain insight [13]. This is essential when handling the complex fax failures in daily practices that usually require human intervention.

To bridge the gap, we developed and configured the BAW platform as the middleware to connect the digital referral platform and the fax receivers. Through integration with the eReferral platform using Health Level Seven (HL7) Fast Healthcare Interoperability Resources (FHIR), the BAW platform will receive the pdf package, including the referral details and the required fax number [14-16]. The BAW platform will then automate the process following a predefined workflow and deliver information to the fax-based receiving clinician through the second integration with the fax service platform. Retry or reminder logic is built into the workflow to increase the success rate of fax delivery.

The Privacy Impact Assessment and Threat Risk Assessment were conducted to ensure the solution complies with Ontario’s Personal Health Information Protection Act and met all privacy and security requirements.

### Workflow Configuration

**Figure 1.**
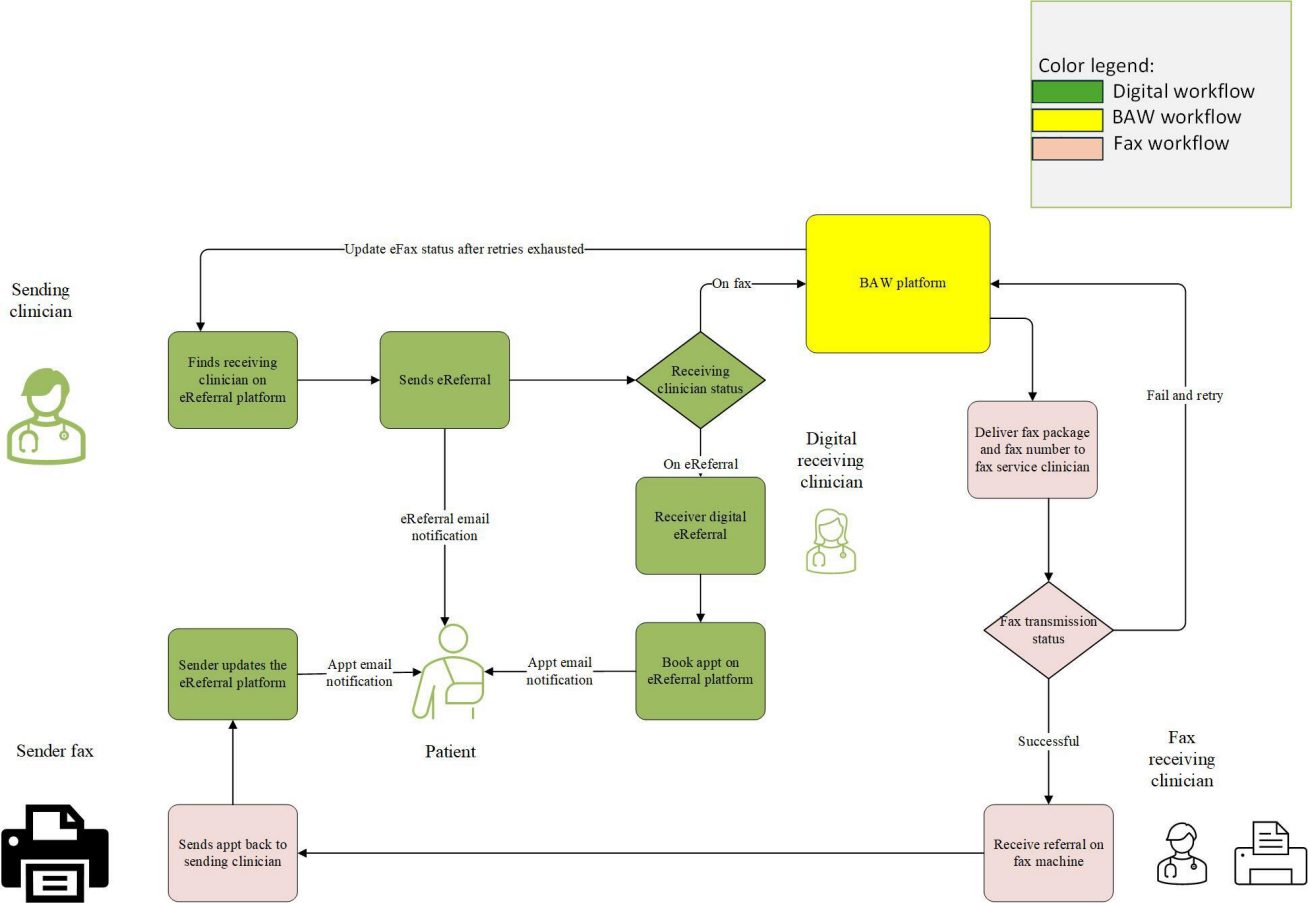
eReferral via eFax workflow diagram. Appt: appointment; BAW: business automation workflow; eFax: eReferral via eFax transaction; eReferral: electronic referral.

The workflow is configured on the BAW platform using business process model and Notation 2.0 to allow the BAW platform to comprehend and follow the defined clinical workflow (Figures1  2). First, the sender starts the eFax using the same workflow directly within the eReferral platform as other digital referrals. Second, the eReferral platform packages the eFax into a PDF document with a cover page and sends it to the BAW platform together with the fax number through the Application Programming Interface. Third, the BAW platform receives the package or correct fax number and passes the information to the fax service following the defined process. Fourth, the fax service will deliver the pdf package to the fax number provided and provide a status update to the BAW platform once the task is completed or failed. Fifth, if the transaction failed, a notification message will be sent to the clinician via the existing eReferral platform to remind the clinician for further action or resubmission according to their discretion. Sixth, retry or reminder logic is included in the workflow to reduce the human interventions required. Seventh, when the patient is booked, the sender receives the response from the receiver using traditional means (fax machine). The appointment details can be entered digitally into the eReferral platform to enable patient notifications so the patient can enjoy the benefits of eReferral technology. Finally, a reminder message will be triggered to the sending clinician if no appointment is booked after a certain period (7 days for an urgent eFax, 30 days for a routine eFax).

**Figure 2.**
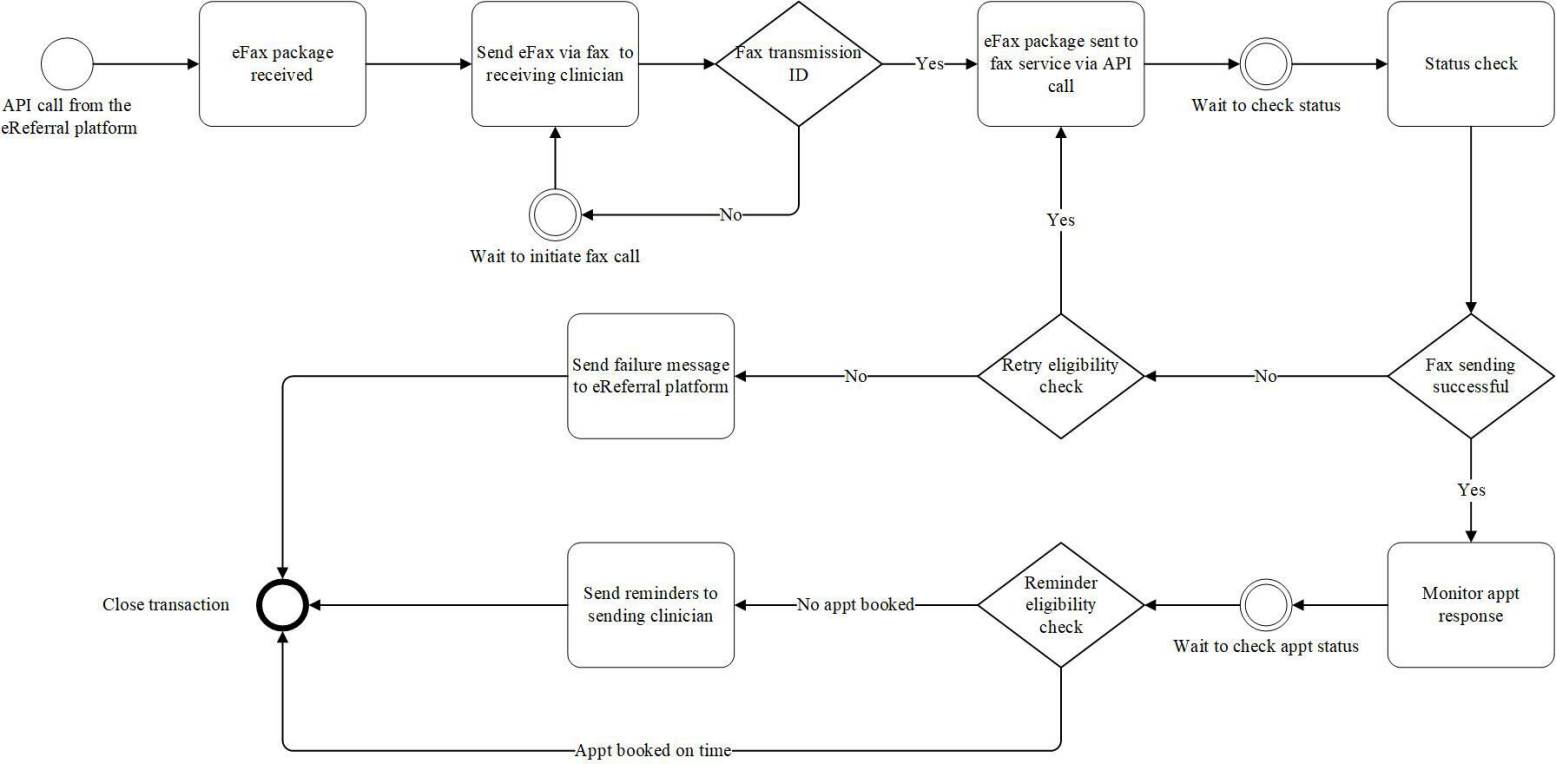
Business automation workflow platform workflow diagram (illustrating retry and reminder logic). API: Application Programming Interface; Appt: appointment; eFax: eReferral via eFax transaction; eReferral: electronic referral.

### Receiving Clinician Recruitment

The PoC started with a select number of clinicians. Working with multiple Ontario Health Teams, the team identified 229 high-volume receivers in the region who have not yet adopted eReferral technology in their practices. Communications were distributed through fax to the identified receivers, and they were provided the choice to “opt-out” if the receiver had concerns about the PoC. A total of 5 receivers reached out and were removed from the list since they were retiring and no longer accepting medical referrals. The rest (224) were successfully added to the referral platform.

### Fax Retry Logic

The PoC was broken into 2 phases to better understand the current fax performance in the Ontario health care system and how the BAW platform can help address the issue. When the fax system communicates with the receiver’s fax system, a status code is generated. The BAW platform then receives this status code and determines if a retry is necessary. The fax failure rate was used to track the fax service performance, which is defined as how often the BAW needs to trigger the retry after the eFax delivery fails. During phase 1 (10 mo, May 2022 to March 2023), the fax-service level retry functionality was disabled to observe the fax performance and understand how frequently the fax process will encounter errors. The BAW platform monitored and tracked every error encountered and the result was analyzed to inform the second phase enhancements. Furthermore, 7 of the most common error codes will trigger automatic retries, including “line busy”, “no answer”, “unexpected disconnect”, “device timeout”, “device busy”, “canceled”, and “no channels available”. The BAW retries once every 30 minutes, up to 10 times, for routine referrals and once every 10 minutes, up to 10 times, for urgent referrals. After all the retries are exhausted, the BAW will mark the referral status as “declined”, attach the reason for the errors encountered, and send the information back to the eReferral platform so the senders can review it for consideration of resubmission.

During phase 2 (March 2023 to December 2023), a new enhanced error handling logic was developed and deployed to retry all error codes and combine the BAW retries with the fax service’s built-in retries to simplify the workflow. The fax service’s built-in retries (every 5 min with total 5 attempts) were enabled to reduce the need for BAW intervention. BAW receives the last status code from the fax service and will trigger a retry during the 5-hour period if the status code is not “no errors.” If the eFax is still not delivered within a 5-hour period, the eFax will be marked as “declined” and returned to the sender for further resubmission.

The retry logic for routine referrals has been updated to retry every 30 minutes during the 5-hour period. For urgent referrals, the BAW system will trigger a retry every 12 minutes.

### Generic Referral Form Development

A working group led by the physician champions was created to discuss the referral forms and templates. Multiple review meetings were held with local clinicians to collect feedback, and a generic eFax referral form template was created and assigned to all eFax referral receivers. The generic form included the option to use a unique form if required by the receiving clinic, which could be included as an attachment[17].

### Evaluation

Solution-generated data were collected from all 3 platforms, including the Business Automation Insights tool (IBM Corp), the eReferral solution, and the fax service portal. The solution-generated data included elements such as fax failure or success rates, page counts, sending and receiving clinician information, referral details like health service offering, referral processing details like status and time stamps, patient email inclusion, and appointment information (if included).

A combination of surveys and interviews collected qualitative data to understand the impact of the eReferral via eFax solution.

Surveys were sent out to all users who have sent or received at least 1 eFax between May 20, 2022 and September 30, 2022. Senders received the survey by email, and receivers received information on how to access the survey by fax. Due to low response rates, the top 10 receivers were contacted by phone to ask if they would participate in the survey. The Ontario Health Teams conducted focus group interviews to gather feedback from eFax senders and receivers. Patient survey data were monitored to see if the satisfaction rate was positively or negatively impacted. Focused interviews were conducted with the eReferral deployment team members to gather their experience onboarding the receivers who transitioned away from eFax to eReferral.

### Ethical Considerations

This study does not require ethics approval as it is quality improvement in nature and uses both publicly available information and secondary analysis with nonidentifiable data as per the Tri-Council Policy Statement from the Government of Canada Panel on Research Ethics [18].

## Results

### eFax Receivers Information

A total of 224 receivers from Ontario Health Regions of Central, Toronto, and West were identified and enabled during the PoC. Of the total, 8 receivers (3.6%) are specialists practicing in academic institutions and hospital clinics, 11 receivers (4.9%) are from specialist group clinics, and the rest (91.5%) are independent specialists. As of December 26, 2023, 14 receivers (6.3%) converted to the eReferral platform (Table S1 in Multimedia Appendix 1). The receiver specialties are primarily in surgical pathways (Table S2 in Multimedia Appendix 1).

### eFax Transaction Information

From May 2022 to December 2023 (as of December 26, 2023), a total of 4504 eFaxes were processed using the eReferral via the eFax platform for 4132 unique patients from 843 unique senders all over the province. Throughout the PoC, 3137 (69.6%) patients received notification emails about the referral details. In total, 875 (19.4%) appointment details were entered into the eReferral platform to support the wait 1 time calculation (Table 1). The receivers declined 158 (3.5%) eFaxes, and the sender entered the decline reason in the eReferral platform with the patient notification message.

**Table 1.** eFax referrals processed by sender region (May 2022-December 2023).

Sender Ontario Health Region	Sender region(historical Local Health Integration Network area)	Referrals, n	Uniquereferrers, n	Unique patients, n	Referral targets, n	Referrals with patient email notification, n (%)	Referrals with appointment booked, n (%)^a^
Central							
	Central	449	110	410	68	337 (75.1)	39 (8.7)
	Central West	152	26	137	56	126 (82.9)	6 (3.9)
	Mississauga Halton	99	33	94	51	62 (62.6)	4 (4)
	North Simcoe Muskoka	16	11	14	14	11 (68.8)	0 (0)
East							
	Central East	92	92	85	51	68 (73.9)	2 (2.2)
	Champlain	6	6	6	6	4 (66.7)	1 (16.7)
	South East	7	6	6	7	4 (57.1)	0 (0)
North							
	North East	8	7	7	8	5 (62.5)	0 (0)
	North West	3	1	3	3	3 (100)	0 (0)
Toronto							
	Toronto Central	448	147	412	93	370 (82.6)	47 (10.5)
West							
	Erie St. Clair	112	36	105	47	57 (50.9)	19 (17)
	Hamilton Niagara Haldimand Brant	1478	107	1361	114	987 (66.8)	453 (30.6)
	South West	1211	164	1114	113	824 (68)	246 (20.3)
	Waterloo Wellington	271	131	247	98	197 (72.7)	40 (14.8)
Total		4504	200	4132	224	3137 (69.6)	875 (19.4)

a Appointment status as of December 26, 2023.

### Fax Performance

The fax performance report is broken into 2 phases.

#### Phase 1

From May 20, 2022, to March 20, 2023, 1706 eFaxes were initiated and processed. As illustrated in Table 2, if the status code indicates “no errors,”, it means the eFax was successfully delivered. If there is a code other than “no errors,” the system counts it as a failure and will start the retry following the retry logic described in the method section. In total, 1658 eFaxes (97.2%) were sent successfully after 0‐10 retries without manual resubmission, and 48 (2.8%) were marked as declined and required manual resubmission from the senders. Among the declined or failed eFaxes, 31 were resubmitted and delivered successfully. The other 17 eFaxes remained in the “declined” folder on the eReferral platform and required sending clinicians’ further attention or resubmission. During phase 1, senders submitted 1706 eFaxes, while BAW made 2712 delivery attempts due to the error codes encountered during the delivery process. Out of these, 1689 (99%) attempts successfully delivered the eFaxes, resulting in an average of 1.59 attempts per successful delivery. The overall fax failure rate was 37.7%. The most common error code is “line busy” (381/2712, 14%), followed by “unexpected disconnect”(224/2712, 8.3%).

**Table 2.** eFax status codes (May 2022-March 2023).

eFax status code	Count, n (%)
Success (no errors)	1689 (62.3)
Line busy	381 (14)
Unexpected disconnect	224 (8.3)
Invalid or missing number**^a^**	183 (6.7)
No answer	150 (5.5)
Error	63 (2.3)
Line failure	10 (0.4)
No dial tone	9 (0.3)
Incompatible fax machine	3 (0.1)
Total	2712 (100)

a This is a system-generated status code indicating there was no valid fax number recognized when communicating with the receiver’s fax machine, although the fax numbers do exist.

#### Phase 2

After March 20, 2023, the new retry logic was deployed to simplify the workflow. From March 21, 2023, to December 26, 2023, a total of 2806 eFaxes were initiated and processed. Of these, 2778 (99%) were successfully delivered to the receivers. Among the delivered eFaxes, 2770 (98.7%) were sent successfully without requiring resubmission, while 36 (1.3%) were marked as declined and required manual resubmission by the senders. Out of the declined eFaxes, 8 were resubmitted and delivered successfully. In contrast, the remaining 28 eFaxes stayed in the “declined” folder on the eReferral platform, needing further attention or resubmission by the sending clinicians.

As illustrated in Table 3, during phase 2, the BAW attempted to deliver 2806 eFaxes, making 3082 attempts in total. Out of these, 2778 (90.1%) attempts successfully delivered the eFaxes, resulting in an average of 1.11 attempts per successful delivery. Under the new retry logic, the fax failure rate, which is the number of times the BAW needs to trigger the retry, decreased to 9.9%. The top error during this period changed to “remote end did not answer” (107/3082, 3.5%), followed by “remote end busy” (89/3082, 2.9%).

**Table 3.** eFax status codes (March 2023- December 2023, post new retry logic deployment).

eFax status code	Count, n (%)
No errors	2778 (90.1)
Remote end did not answer	107 (3.5)
Remote end busy	89 (2.9)
Unexpected error	72 (2.3)
No fax was detected	25 (0.8)
Unable to negotiate fax session with remote terminal	7 (0.2)
An error occurred during the file rasterization process	2 (0.1)
Failure to confirm page reception by remote terminal	1 (0)
Remote terminal terminated the fax session unexpectedly	1 (0)
Total	3082 (100)

### Referral Pages Count

**Figure 3.**
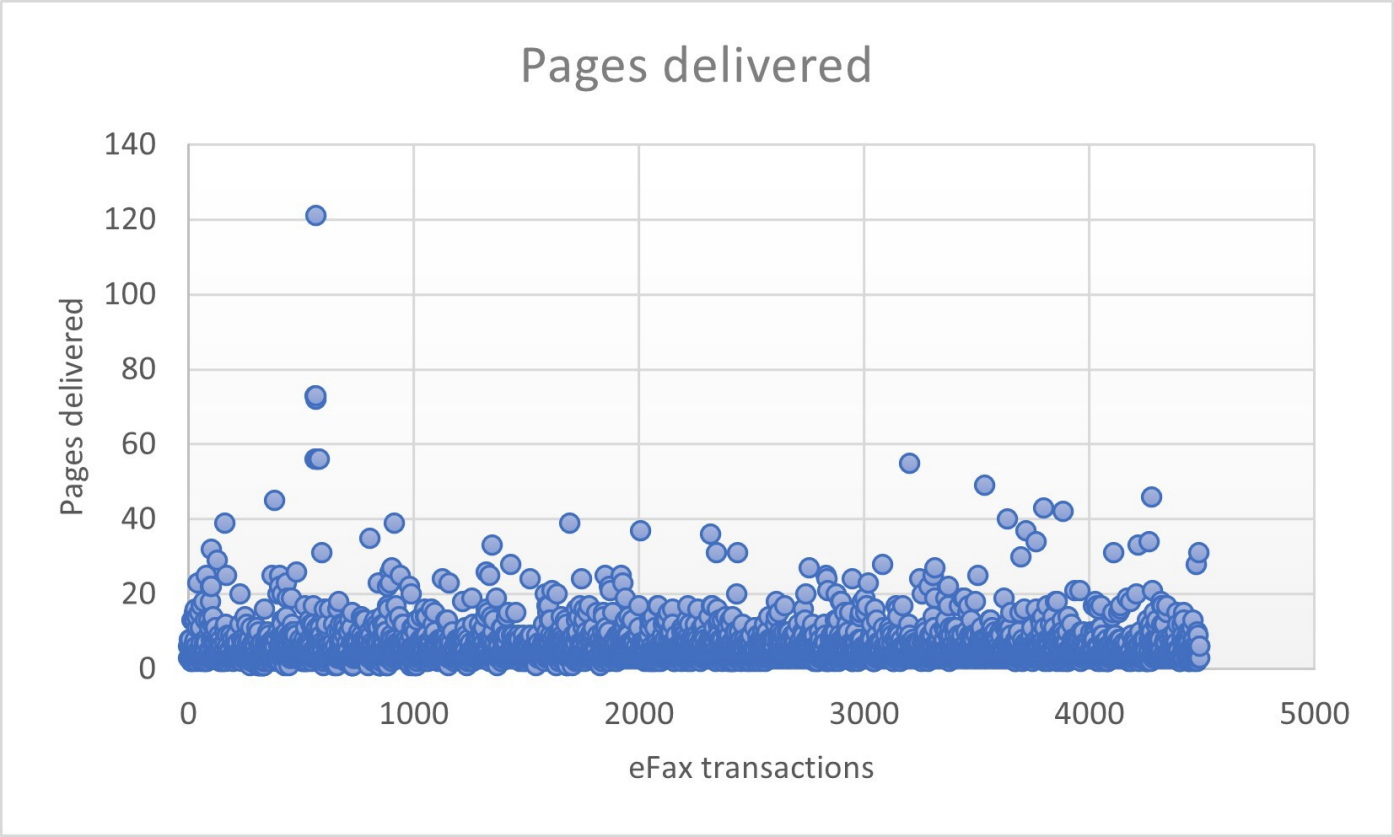
eFax pages delivered scatter plot diagram.

The majority of eFaxes processed during the PoC were between 1 and 10 pages long (4115/4504, 91.4%), with the most common number of pages being 3 (1951/4504, 43.3%), and an overall average of 5.6 pages per eFax. A few outliners were observed, such as 32 eFaxes (0.7%) having more than 30 pages in the packages, with a maximum of 121 pages (Table 4 and Figure 3).

**Table 4.** eFax pages processed count.

Pages delivered	Transactions count, n (%)
1	38 (0.8)
2	151 (3.4)
3	1951 (43.3)
4	416 (9.2)
5	517 (11.5)
6	335 (7.4)
7	281 (6.2)
8	201 (4.5)
9	138 (3.1)
10	87 (1.9)
11‐20	307 (6.8)
21‐30	50 (1.1)
31+	32 (0.7)
Total	4504 (100)

### Clinician and Patient Experience

During the PoC, the primary care sender survey was sent to 147 unique sending clinicians, and responses were received from 18 (12% response rate). Of the 18 responses, 72% (n=13) were satisfied with the eFax workflow, and 72% (n=13) felt the eFax has improved their ability to track or follow the referrals (Multimedia Appendix 2).

Multiple senders still noted that there were not enough receivers on the map. Furthermore, 4 out of the top 10 receiver clinics were able to answer a phone call from the PoC team, but none of them were aware of the eFax PoC work, although they did receive the most eFaxes during the PoC and multiple faxes were sent to their clinics. They did not notice the difference between the eFaxes and their regular fax-based referrals. The findings from the focus group interviews illustrated that the primary care senders felt that the new solution improved their workflow, helped them track the referral status, and that patients were more involved in the process. On the other hand, receivers noticed the eReferral platform logo, but there was no positive or negative impact on their existing workflow. Patient experience data related explicitly to the eFax referrals processed over the evaluation period could not be segmented from the eReferral dataset. However, patient satisfaction rates remained consistently high during the PoC time, similar to those seen before the PoC was implemented, which could be an indicator that from patient perspective, satisfaction with the referrals made via eFax was high and similar to the satisfaction with eReferral. However, more data will need to be collected to prove this assumption. Feedback from interviews with 4 different deployment team members indicated that there was no significant difference in engaging the eFax receivers as compared with the routine flow when onboarding as an eReferral receiver.

## Discussion

### Study Rationale

Ontario has made several efforts to improve patient information sharing in clinical settings. The Ontario Medical Association has recommended transitioning from fax technology to the eReferral system to enhance efficiency and patient experience [5]. The Ontario eServices program was established to provide change management support and expedite the adoption of eReferral technology. Digital standards, such as the digital health information exchange framework and eReferral HL7 FHIR specifications, have been established to improve interoperability and secure information sharing [16]. In addition, the government has initiated a plan to phase out fax machines by 2028, promoting the adoption of digital communication alternatives [11]. While transitioning to eReferral offers numerous benefits, a significant barrier to its adoption is the requirement for both sending and receiving clinicians to be on the same platform for effective information sharing. The traditional onboarding process is time-consuming and labor-intensive. Therefore, an innovative method is essential to bridge the gap between digital eReferral senders and fax-based referral receivers, thereby expediting eReferral adoption.

This PoC study investigated the feasibility of using the BAW platform as a temporary solution to support eReferral adoption by connecting the eReferral platform and select fax services, enabling them on the eReferral platform as eFax receivers. This solution aimed to improve the sending clinicians’ experience by expanding the available receiving clinicians on the platform, reducing workflow redundancy and leveraging the BAW platform to facilitate the senders’ transition to eReferral. The solution is designed to provide a seamless user experience and can be used as a temporary bridging technology while the onboarding process is underway.

### Clinician Experience Improvement

The solution design principle focused on minimizing the impact on the current workflow for both senders and receivers. Senders can reduce the need to maintain 2 separate workflows for eReferral and non-eReferral receivers, accessing an expanded list of eReferral receivers. eFax receivers can keep their current workflow with no alterations, as the referrals made to them look the same as fax referrals and do not require any workflow changes. The survey findings reflected that the clinicians felt the solution achieved the goals of the design principle; the senders were satisfied and they felt it improved their workflow while receivers’ workflows were not interrupted and they noticed minimal changes throughout the PoC. The only behavioral change required was the need for senders to manually enter the appointment information back into the eReferral platform so that the patient can be notified through emails. Due to the extra tasks required, the entry percentage was 19.4% for the eFaxes booked (Table 1) with appointment dates documented in the eReferral platform, as compared with 57% of eReferrals within that same time period. As such, there is an opportunity for future enhancements to better capture the appointment information for eFaxes sent. Although this was lower than the automatic collection via the traditional eReferral workflow, this still presented an opportunity to calculate the wait time information so it can be published for eFax receivers, supporting clinician informed decision-making when initiating a referral for a patient.

In the current hybrid workflow, a sender needs to go back to the fax referral workflow if the receiver is not on the eReferral platform. Senders either need to maintain the duplicate workflow or prompt the receivers to adopt the eReferral technology, which usually takes a few weeks after the receivers agree to participate. By bridging the gap with the BAW platform, senders can ensure that they take advantage of the benefits of eReferral without experiencing any significant disruptions or delays. The enabled eReferral via eFax receivers list was expanded to allow eReferral to flow to the receivers unavailable through the traditional eReferral workflows. This solution allows for the eReferral platform to have more transactions flow through the solution reducing the need for eReferral users to maintain more than one referral pathway in order to provide care to their patient population. The more receivers are enabled through this solution, the less the need for senders to fax referrals, with the optimal goal to have senders send all referrals through a similar workflow, electronically.

### Patient Experience Improvement

It is reported that eReferral can improve patients’ experience with the referral process, and more specifically that 80% of patients felt they were more informed during the referral process [4]. The eReferral via eFax technology enables the patients to experience the benefits of eReferral even though the receivers were not onboarded to eReferral. Throughout the PoC, 3137 (69.6%) patients had their email included in their referral and received notification emails about the referral progress and updates. eFax specific patient experience survey results could not be segmented from the eReferral data set during the same period and therefore could not be assessed. However, the overall patient satisfaction rate remained consistently high throughout the PoC period.

This technology enables the option of patients being included in the communication loop on their referrals, regardless of whether the receiver of their referral is on fax or eReferral, as the solution enabled email notifications, keeping patients informed surrounding referral status updates, throughout the referral journey. This technology reduces the known issue for patients, as traditional fax referrals can never send an email notification to patient. This can contribute to fulfilling the commitment of patient-centered care, even during this migration phase.

### Cost Saving

The new bridging technology also helps to save costs. First, the technology reduces senders’ need for paper usage, as they no longer need to print and fax documents. This can lead to significant cost savings. During the PoC, the average number of pages sent was 5.6 pages, translating to about 11 pages for both senders and receivers combined. It is reported that primary care clinicians send a median of 26 referrals per month [19]. The bridging technology can reduce half of that cost through the adoption of eFax and eventually remove all the paper costs when all the senders and receivers are onboarded to the eReferral platform. In addition, costs related to fax maintenance and printing ink would significantly be reduced over time.

Second, as part of the eReferral technology benefits, the eFax technology triggers email communication for patients, which can help avoid staff calls to the primary care clinic and reduce the time patients and clinical staff spend hunting down information [20-23]. This can lead to significant time savings for both patients and clinical staff. The new bridging technology is a step toward a more efficient and cost-effective health care system.

Third, the built-in BAW retry logic helped to reduce the human effort required to track the fax status and resend if the fax fails. The data from both phases illustrated in real practice when using the fax technology to communicate, the clinicians still need to manually monitor and resend the fax if the fax fails. Failures occurred in 37.7% (1023/2712) of transmissions without the fax service retry enabled (phase 1), compared with 9.9% (304/3082) with the retry enabled (phase 2). During phase 1, the BAW platform automatically retried failed eFax transactions to improve the delivery successful rate to 97.2%, avoided 37.5% (1017/2712) manual resubmission, and only 2.8% (48/1706) eFaxes required manual intervention. During phase 2, after the fax service automatic retry setting was enabled, the fax failure rate was reduced to 9.9%, which still required the built in BAW retry logic to automatically resend the failed eFaxes. The new updated retry logic improved the delivery success rate to 98.7% and further reduced the manual monitoring and resubmission to 1.3%.

There were improvements made in the eFax technology, and the BAW retry logic that reduced the failure rate but there is still increased value in transitioning to eReferral. eReferral requires extremely few retires, if any, due to the digital nature of the system. Unless there is a system or internet connectivity disruption, the eReferral will be delivered to the receiver immediately upon sending. There will be less failures and improved connectivity between the senders and receivers, limiting the risk of lost or failed referrals and decreasing the negative outcomes for the patient that might occur due to failures or lost transmissions.

### System-Level Benefits

The eReferral via eFax platform provides system-level benefits that are only available using the eReferral platform and are otherwise unavailable in traditional nondigital workflows. In the fax referral workflow, transaction data are often recorded on paper, making tracking the referral flow from senders to receivers difficult. In addition, the wait times of receivers are generally not publicly available since it is challenging to track when and how appointments are booked. By leveraging the BAW platform, transactional data can be accessed earlier, and insights into the referral process can be gained that would otherwise be impossible. By providing visibility into the referral flow, the BAW platform can help optimize the referral workflows and improve the patient’s experience. The BAW platform can also help identify bottlenecks in the referral process and take corrective action to improve the efficiency of the process. When the senders make referrals, essential factors like wait times and distance can be reviewed to support a more informative decision-making process, supporting better outcomes for patients.

The traditional onboarding process of an eReferral receiver includes multiple steps, such as the initiation of the discussion, referral form review and customization, EMR integration deployment, and end user training. Based on the learnings from the Ontario eServices Program, this process usually takes 6‐8 weeks of change management support and sometimes even longer. Compared with the traditional receiver onboarding process, enabling the eFax workflow only includes receiver identification and validation, which usually takes a few minutes. The new process can drastically reduce the time required for the receiver onboarding process. The senders can start having a broader receiver list on the eReferral platform without disrupting the regular receiver onboarding process. It is very common for the senders to send referrals across regions (Table 1), and enabling the receivers through the proposed technology has the great potential to allow a large number of receivers enabled across the regions. Meaning, more receivers (as well as their wait times) will be visible on the eReferral platform to support the senders’ decision making. It is beneficial to use relatively less effort to allow the maximum benefits that both senders and patients can start to experience. At the same time, the normal eReferral onboarding process can still take its own pace while increasing the number of receiving sites to support increased usage by those senders already within the eReferral network. The senders and their adoption of eReferral are currently restricted by the number of receivers present on the eReferral platform and users note that expanding the receiver list increases their usage of the tool. The lack of having a sufficient number of receivers that eReferrals can be sent to could lead to less opportunities to send eReferrals for clinicians and thus become a risk of discontinuing eReferral use by maintaining one workflow via faxing. This may lead to the need of re-engaging of clinicians to promote active use of eReferral, which in turn presents a monetary loss for the health system.

### Future Work

There are more than 17,800 specialists registered in Ontario that might receive referrals [24]. With other hospital service providers and independent Health Facilities, Ontario’s total possible medical referral receivers are likely more than 19,000. The Ontario eServices Program (previously System Coordinated Access) started to onboard clinicians to the eReferral platform in 2017. Using an approach that focused on both the onboarding of primary care clinicians as well as receiving specialists, in just over 6 years, in Ontario 2000 receivers and 11000 senders were onboarded with change management supports, accounting for over 2 million referrals processed. There is still significant progress to be made. The eServices Program has been working on multiple strategies to increase the speed of adoption, such as exploring the use of the central intake models. A more innovative solution may be required to support eReferral adoption compared to the number of possible receivers.

The BAW platform could be used to bridge the gap between the fax system, that is widely used in the Canadian health care system, and the new eReferral systems, allowing minimal disruption of the sender’s workflow while receivers using the fax system can take their pace being onboarded to the new platform. This approach presents a significant value for senders to adopt the eReferral solution, as it can provide access to a large number of referral receivers from the start of the initiative, which it can be difficult to attain through eReferral onboarding and change management initiatives alone. Senders may not wish to adopt the solution if it does not allow them access to a large number of receiving specialists, and specialists may not want to adopt the solution and introduce another workflow in their clinic as there may not be a sufficient number of primary care clinician senders onboarded on eReferral to send eReferrals to them. This presents a challenging dilemma on which to focus first, that can be minimized with the BAW platform allowing access to large number of receivers with minimal effort, presenting a favorable context for sender onboarding, followed by receivers.

The current evaluation demonstrates the effectiveness of expediting eReferral usage through the eReferral via eFax platform, while also highlighting the potential to further increase utilization by including additional receivers. In addition, there is a future opportunity to conduct a time-saving study to illustrate further the cost savings achieved by implementing this methodology.

## Supplementary material

10.2196/62983 Multimedia Appendix 1 eFax receivers distribution.

10.2196/62983 Multimedia Appendix 2 Primary care sender clinician survey results.

## Abbreviations

BAW: business automation workflow
EMR: electronic medical record
eReferral: electronic referral
FHIR: Fast Healthcare Interoperability Resources
HL7: Health Level Seven
NHS: National Health Service
PoC: Proof-of-Concept
