# Supplementary material for: A Unique Way to Axe the Fax Through Using Business Automation Workflow to Expedite eReferral Adoption, Bridging eReferral, and Fax: Proof-of-Concept Study
Source: JMIR Med Inform. 2025 Jun 30;13:e62983. doi: 10.2196/62983 (PMC12234396; doi:10.2196/62983)
Supplement: Multimedia Appendix 1 [file medinform-v13-e62983-s001.docx]

**Table S1.** Receivers onboarded and converted.

| Receiver | OHT^a^ 1 | OHT 2 | OHT 3 | Total |
| --- | --- | --- | --- | --- |
| Total number of receivers onboarded | 71 | 103 | 50 | 224 |
| Number of receivers converted to eReferral^b^ (to date) | 5 | 9 | 0 | 14 |

^a^OHT: Ontario Health Team.

^b^eReferral: electronic referral.

**Table S2.** eFax receiver specialties.

| Specialty | Numbers of receiving clinician |
| --- | --- |
| Obstetrics and gynecology | 32 |
| Orthopedics | 29 |
| Gastroenterology | 21 |
| General surgery | 21 |
| Urology | 11 |
| Allergy and immunology | 10 |
| Dermatology | 10 |
| Cardiology | 9 |
| Rheumatology | 8 |
| Otolaryngology | 8 |
| Fertility | 7 |
| Plastic surgery | 7 |
| Endocrinology | 6 |
| Pediatric gastroenterology | 5 |
| Nephrology | 5 |
| Neurology | 5 |
| Pediatrics | 5 |
| Ophthalmology | 4 |
| Pediatric allergy | 4 |
| Sports medicine | 3 |
| Dermatology-pediatrics | 2 |
| Breast disorders | 2 |
| Hematology | 2 |
| Infectious diseases | 2 |
| Respirology | 2 |
| Head and neck | 1 |
| Geriatric medicine | 1 |
| Thrombosis | 1 |
| Psychiatry-pediatrics | 1 |
| All | 224 |
