# Supplementary figures and images for: A Unique Way to Axe the Fax Through Using Business Automation Workflow to Expedite eReferral Adoption, Bridging eReferral, and Fax: Proof-of-Concept Study
Source: JMIR Med Inform. 2025 Jun 30;13:e62983. doi: 10.2196/62983 (PMC12234396; doi:10.2196/62983)

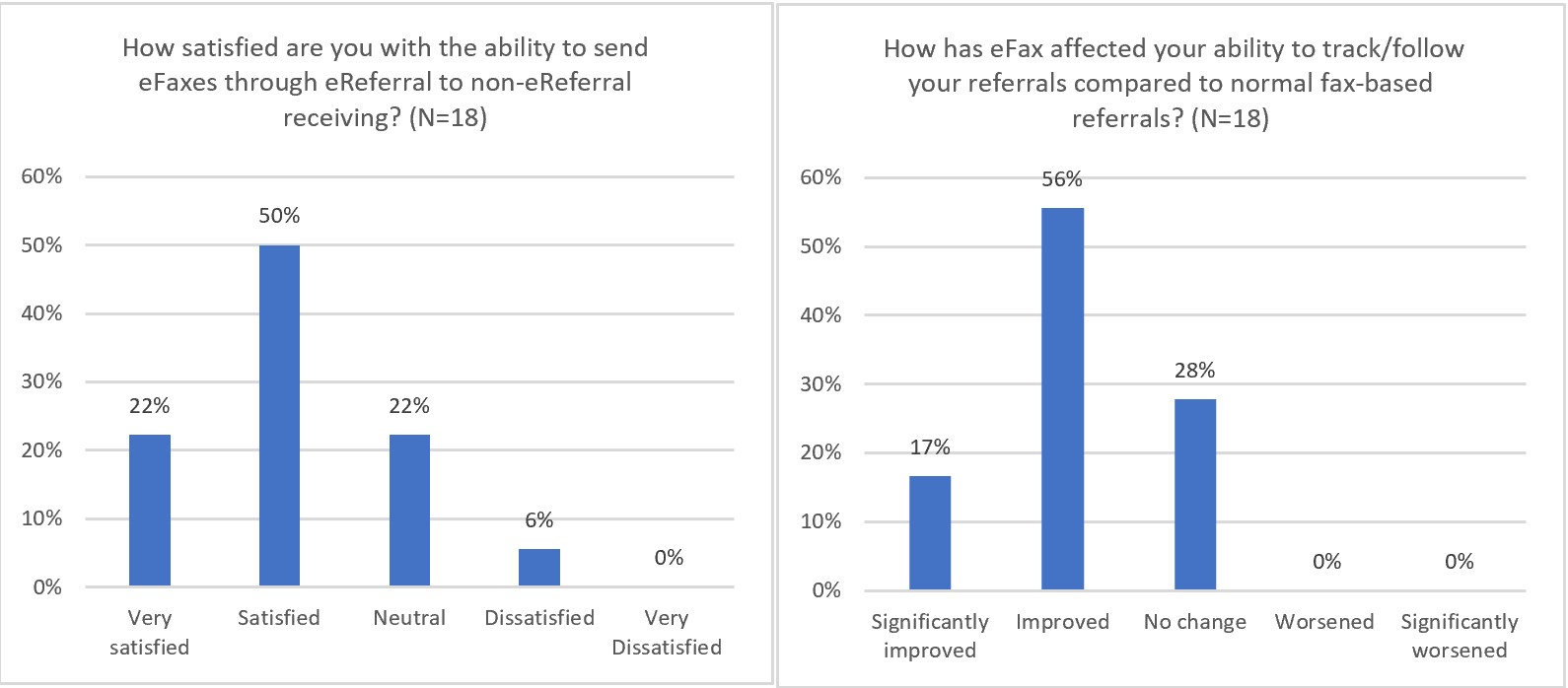

Supplement: Multimedia Appendix 2 [file medinform-v13-e62983-s002.jpg]
